# Attentional Demands of Movement Observation as Tested by a Dual Task Approach

**DOI:** 10.1371/journal.pone.0027292

**Published:** 2011-11-03

**Authors:** Cinthia M. Saucedo Marquez, Tanja Ceux, Nicole Wenderoth

**Affiliations:** Group Biomedical Sciences, Department of Biomedical Kinesiology, Research Centre for Motor Control and Neuroplasticity, K.U.Leuven, Heverlee, Belgium; University of Bologna, Italy

## Abstract

Movement observation (MO) has been shown to activate the motor cortex of the observer as indicated by an increase of corticomotor excitability for muscles involved in the observed actions. Moreover, behavioral work has strongly suggested that this process occurs in a near-automatic manner. Here we further tested this proposal by applying transcranial magnetic stimulation (TMS) when subjects observed how an actor lifted objects of different weights as a single or a dual task. The secondary task was either an auditory discrimination task (experiment 1) or a visual discrimination task (experiment 2). In experiment 1, we found that corticomotor excitability reflected the force requirements indicated in the observed movies (i.e. higher responses when the actor had to apply higher forces). Interestingly, this effect was found irrespective of whether MO was performed as a single or a dual task. By contrast, no such systematic modulations of corticomotor excitability were observed in experiment 2 when visual distracters were present. We conclude that interference effects might arise when MO is performed while competing visual stimuli are present. However, when a secondary task is situated in a different modality, neural responses are in line with the notion that the observers motor system responds in a near-automatic manner. This suggests that MO is a task with very low cognitive demands which might be a valuable supplement for rehabilitation training, particularly, in the acute phase after the incident or in patients suffering from attention deficits. However, it is important to keep in mind that visual distracters might interfere with the neural response in M1.

## Introduction

Movement Observation (MO) activates the motor system of the observer in a similar way as movement execution. This was demonstrated at the single cell level in monkey's inferior frontal and inferior parietal cortex where so called “mirror neurons” fire when an action is performed but also when the same action is merely observed [1]. In humans, MO activates the same motor areas [2]–[7] as movement execution and, particularly, modulates the corticomotor excitability of the primary motor cortex (M1) as measured by transcranial magnetic stimulation (TMS). M1 facilitation is muscle and force specific [8]–[15] that follows the timing of the observed action [16] and is influenced by posture and perspective [17]–[19] leading to the view that the observer's motor system directly matches the perceived action to the corresponding motor representation. Mainly based on two different research lines, it has been suggested that the direct matching mechanism during MO is a “near-automatic” process with low attentional costs [20]–[23]. First, functional imaging results demonstrated that MO activates motor areas to a similar extent when subjects either focus on the displayed actions or divide their attention between the movement stimuli and a secondary attention demanding task [24]. Second, behavioral studies showed consistently that responses to stimuli depicting motor actions are faster when the executed and observed movement are congruent than when they are incongruent [25]–[33]. This so-called “automatic imitation” effect was demonstrated even when the observed action was irrelevant to the participants' response or when participants attended to an orthogonal stimulus dimension (e.g., to the brightness of the shown limb than to the observed movement, [32]).

Recent behavioral studies using a similar reaction time paradigm, however, revealed inconsistent results. Bach et al. (2007) showed whole-body pictures of an actor as imperative cue and found that the congruency advantage was only present when subjects attended to the body part relevant for the reaction time task (e.g., attending to the actor's foot when responding with the foot), but not when attending to a neutral body part (e.g. the actor's head). Similarly, Chong et al (2009) reported that the congruency advantage of action stimuli was abolished when the participant's attention was directed away from movement features, e.g., when response selection depended on a symbolic cue not related to motor actions such as the color of a symbol overlaying a snapshot of a hand action. These latter behavioral results are in conflict with the automatic imitation hypothesis and suggest that MO is influenced by attentional top-down control even when the action stimuli are simple and easy to identify. Also at the neural level it was shown recently that brain activity within classical MO areas is enhanced when subjects allocate attention to the movement stimuli [25], [34] a finding that is inconsistent with Jastorff et al (2010) who reported similar brain activation when movement observation was performed as a single or a dual task. The results of Chong et al. (2009) and Muthukumaraswamy and Singh (2008) suggest that brain activity during MO is modulated by attention, however, it has to be noted that this result does not allows to draw firm conclusions about the *attentional costs* related to MO.

To answer this question we used TMS to measure changes in corticomotor excitability of the observer's primary motor cortex (M1) while participants observed simple motor actions either alone (single task) or in parallel with a demanding secondary task (dual task). During the observation task, participants watched how an actor grasped and lifted either a light or a heavy object. Based on previous research [9], [10] we predicted that for the single task condition, M1 excitability would be modulated in accordance to the weight of the object indicative of a direct observation-execution matching mechanism potentially mediating action understanding. Critically, we tested whether M1 excitability would exhibit a similar weight-related modulation when subjects performed a demanding discrimination task in parallel, suggesting that this response is evoked by a near-automatic mechanism. This dual task approach assumes that attentional control imposes restrictions when two tasks are executed in parallel as conceptualized by the central capacity sharing model [35]. The central capacity sharing model proposes that dual task interference arises due to capacity limitations [35]. Thus, when two tasks are performed in parallel, the total capacity will be divided and processing will occur simultaneously. In this case, performance deterioration due to dual task interference will arise when competing task demands exceed the available computational resources [36]. Interestingly, the central capacity sharing model predicts less dual task interference when two tasks access different sensory modalities, as each modality is entitled to its own separate attention resources [37].

Here we test this prediction by instructing subjects to perform the movement observation (MO) task together with either an auditory or a visual discrimination task. We hypothesize that modulations of M1 excitability in relation to force requirements of the observed action reflect a direct observation-execution matching mechanism during MO. If this MO specific modulation is absent during the dual task condition it signals interference and one would conclude that MO requires at least some attentional resources. Moreover, if the modulation of M1 excitability is specifically perturbed when the secondary task accesses the visual domain, it would suggest that MO relies specifically on visual attention.

Further insights into the attentional demands of MO are not only of academic interest but might also have clinical implications, as MO might be an additional form of therapy, for example after stroke [38]–[40] that can be used in patients with attentional deficits.

## Methods

### Ethics Statement

Study protocol and informed consent were approved by the local Ethics Committee for Biomedical Research at the Katholieke Universiteit Leuven and in agreement with the Code of Ethics of the World Medical Association (Declaration of Helsinki) [41]. Written informed consents were obtained from all subjects.

### Subjects

Fourteen subjects (8 females, age 20.6 yrs±2.4 yrs) participated in experiment 1 and a different group of twelve subjects (4 females, age 22.8 yrs±3 yrs) was included in experiment 2. All participants were self-reported right hander's, wrote with their right hand and had high positive scores in the Oldfield Questionnaire [42] (Exp 1: ranging from 76 to 100, mean score 95±10,34; Exp 2: ranging from 41 to 100, mean score 96±10). Everybody was naive to the purpose of the study and had no overt sensorimotor or neurological deficits. Each participant was screened for risk factors and potential adverse effects caused by TMS and signed an informed consent before the experiment. The results of one participant from experiment 1 were excluded from further analyses due to lack of obedience to perform the task as instructed.

### Procedure of measurements

The following TMS protocol was identical for both experiments. TMS was delivered via a Magstim 200 Stimulator (Magstim, Whitland, Dyfed UK), connected to a figure-of-eight coil (70 mm diameter) to deliver focal TMS pulses. The coil was positioned over the left hemisphere such that the handle pointed away from the midline by a 45° angle. This position ensured a posterior-lateral to anterior-medial flow of the induced current, approximately perpendicular to the central sulcus, which is optimal for stimulating the corticospinal pathway of M1. Motor Evoked Potentials (MEPs) were recorded by means of an electromyogram (EMG) which was measured by two disposable Ag-AgCL surface electrodes (Blue sensor SP) placed over the opponens pollicis (OP) muscle in a belly-tendon montage. The OP was chosen because it is strongly involved in grasping as shown in the videos and is facilitated in a weight-specific manner during movement observation [9]. The responses were sampled at 5000 Hz (CED Power 1401, Cambridge Electronic Design, UK) amplified, band-pass filtered (5–1500 Hz), and stored for offline analysis. The EMG signal was displayed online and visually inspected for increased background activity.

TMS was used to determine the so called “hotspot”, i.e., the position where MEPs with the highest and most consistent amplitudes were evoked in the right OP muscle. At the hotspot, the Rest Motor Threshold (RMT) was determined as the lowest stimulation intensity to evoke MEPs of at least 50 µV in 5 out of 10 consecutive stimulations [43]. During the experiment, subjects were stimulated at 130% of their individually determined RMT. Pre-stimulus EMG activity was quantified by the Root Mean Square Error (RMSE) calculated within a 100 ms interval preceding the magnetic pulse (110–10 msec. before TMS) and used to assess the presence of unwanted background EMG activity. TMS triggering and EMG recordings were controlled by Signal Software (2.02 Version, Cambridge Electronic Design, UK).

### Design

The participants were seated in front of a digital computer screen (Dell 1707, resolution 1152×870 pixels, refresh frequency 60 Hz), at a distance of approximately 50 cm, with their hands out of sight and supported by a soft cushion to ensure relaxation. They were instructed to keep their forearm and hand muscles as relaxed as possible and the background EMG activity was monitored by the experimenter.

**Figure 1:**
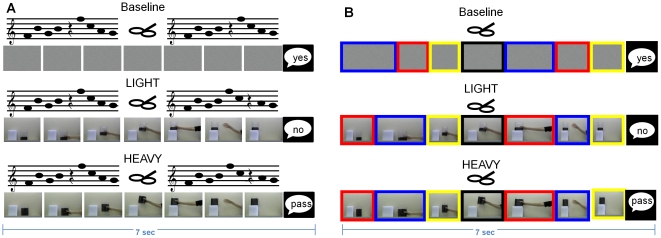
Video clips and discrimination tasks of experiments 1 and 2. Both experiments used the same 3 different video clips: baseline (top), light (middle) and heavy (bottom). Movement observation was presented together with an auditory discrimination task (**A**, experiment 1) or a visual discrimination task (**B**, experiment 2). The six different video sequences presented here represent one single trial with duration of 7 sec each. **A** In experiment 1, subjects had to discriminate two series of tones such that the position of one longer inter-tone-interval could either be identical (**A,** top) or different (**A**, middle). B In experiment 2, subjects had to discriminate to series of color changes of the rim. One of these colors was shown longer (as indicated by wider rectangles), however, the rhythm of the color change could either be identical (**B**, top) or different (**B**, middle). In both experiments, subjects had to say “yes” when the two series of tones/colors were identical, “no” when they were not identical, and “pass” when no decision could be made. The verbal response was provided after the video clip had finished. TMS was applied in between the two series of tone, when the object was lifted in the air, as symbolized by the TMS coil.

Three different digital videos of grasping actions were shown in a randomized order (Fig. 1A): 1. A static scrambled image of the general screen (baseline) 2. A hand that enters the scene grasps and lifts a light object to place it on an elevated position (LIGHT). 3. A hand that enters the scene, grasps and lifts a heavy object, and places it on an elevated position (HEAVY). All video clips were shown in the sagittal plane and the actor used a whole hand grip to lift and place the object (Fig. 1). The video clips were displayed with a frame rate of 25 Hz, each clip lasted 6 s separated by 1 sec. breaks (black screen) resulting in a total duration of 7 sec forming a trial (Fig. 1). Each trial was repeated 5 times forming one block and TMS was applied once for every trial (i.e. one block lasted 35 sec and 5 TMS pulses were recorded). This “block design” was applied because it reveals consistent MEP responses as demonstrated previously [8]–[10], [18], [44].

Subjects were instructed to watch each trial attentively and to report after the 35 sec. block whether the object lifted in the 5 trials was light or heavy. Note that this task was very undemanding and that it was easily performed by the subjects. The digital video clips showing the motor actions were identical in both experiments, however, the discrimination task accessed either the visual or the auditory modality.

In experiment 1, subjects solved an auditory discrimination task. Subjects had to discriminate between two series of tones, each consisting of 8 tones with different pitch (400–600 Hz, duration 0.1 sec.). Seven tones were played with a short inter tone interval of 0.165 sec, and one with a long inter tone interval of 0.33 sec. The two series of tones were either identical or differed because the long inter-tone interval was shifted to another position (see Fig. 1). The two series of tones were preceded by a voice saying “one” and “two”, respectively and they were separated by silence lasting 1 sec. After the second series of tones, when the 7 sec trial has ended, subjects had to answer whether the two series were identical (“yes”) or not (“no”) or whether no decision could be made (“pass”). The tone discrimination sequences were randomly assembled by the acquisition software which also assured that sequences were identical in 50% of all trials.

Importantly, the auditory stimuli as well as the verbal responses of the subjects were kept identical in all conditions, i.e., subject watched always one of the three video clips while hearing the two series of tones and always had to give a verbal response. In the single task condition, subjects were instructed to pay attention to the video shown in the trial while ignoring the tones and to answer “yes” by default. In the dual task condition, subjects had to pay attention to both the video shown in the trial and the auditory discrimination task. In all conditions the TMS timing was adjusted such that the stimulation was applied in the break between the two series of tones when the object was lifted in the air.

In experiment 2, a visual stimulus was added to the video shown in each trial; such that the rim around the video changed colors every 200 or 750 msec. Participants were asked to discriminate between two series of three colors separated by a black colored rim (see Fig. 1). The order in which the colors were presented was the same in both series. However, each color was shown either for 200 or 750 msec. and the timing could differ between the two series. For example, the first series might be yellow (200)-red (750)-yellow (200) and the second yellow (200)-red (200) yellow (750). After the second series of colors was observed i.e. during the break between two video clips, subjects had to answer whether the two series of colors were identical (“yes”) or not (“no”) or whether no decision could be made (“pass”). Also the color discrimination sequences were randomly assembled and the stimuli were kept constant across conditions, i.e., the tones/changing colors of the rim were always present and subjects were instructed to focus on the grasping videos only (single task condition) or to perform the auditory/visual discrimination task in parallel (dual task condition). Hence, in the single task condition subjects had to give a verbal response (“yes” by default) at the end of each trial (Fig. 1). In the dual task condition for experiment 2, they were instructed to look at the action stimuli and solve the color discrimination task via peripheral vision. In all conditions the TMS timing was adjusted such that the stimulation was applied in the break between the two color series when the object has been lifted by the actor.

In both experiments, there were 6 different conditions (baseline, LIGHT, HEAVY movie watched either under single or under dual task condition). For each of these conditions the same video was shown 5 times (forming one block where 5 MEPs were collected) while the auditory/visual discrimination task changed continuously. We tested 4 blocks for each condition resulting in 20 trials/MEPs per condition (120 in total). Breaks were allowed whenever needed by the subject. Importantly, the condition blocks were presented in pseudorandom order across subjects. More specifically, we presented all 6 condition blocks in random order and repeat this procedure 4 times (each time the order of the 6 condition blocks was randomized). A typical randomization for one subject would be: 4 2 3 5 1 6 2 1 5 6 4 3 6 4 3 2 1 5 3 5 6 4 2 1 (with numbers indicating the different conditions). Note that randomization was different in each subject to exclude order effects. We chose this manner of pseudo-randomization for two reasons: Fist of all, it minimizes order effects at the group level. Secondly, it is possible that responsiveness to TMS changes slightly during an experiment. By cycling through all 6 conditions within each of the 4 repetitions the direct comparison between conditions is relatively close in time and direct comparisons are less confounded by a slow excitability drift.

After the experiment, subjects were de-briefed and all reported that they experienced discrimination task, and particularly the auditory version, as challenging.

### Data analysis

All trials were visually inspected and trials with increased pre-trigger EMG activity (RMSE >0.004 mV) were removed from further analysis (13.1±9.8%). For the remaining trials, the peak-to-peak amplitude of the MEPs was determined as an index of the corticomotor excitability of the OP. MEP amplitudes were averaged within each condition, and a z-transformation was applied to reduce inter-subject variability which can be substantial when absolute MEP size is used.

### Statistics

The z-transformed mean MEP amplitudes (zMEPamp) and the pre-trigger EMG as quantified by the RMSE were subjected to separate two-way Analysis Of Variance models for repeated measurements (ANOVA) with the within subject factors “task” (single, dual) and “movie” (baseline, LIGHT, HEAVY). Pre-planned comparisons were used to test specifically whether zMEPamp were larger for the HEAVY than the LIGHT condition, as predicted by previous data [9], [10].

Discrimination performance (% correct answers) of the secondary tasks was subjected to one-way repeated measures ANOVA to compare discrimination performance between baseline, LIGHT and HEAVY. The discrimination results of two subjects from experiment 1 were lost due to technical malfunction.

Finally, the judgment of the object's weight lifted in the video (% correct answers) was compared between single and dual task conditions by a t-test. For all statistical tests, the α-level was set to α = 0.05. Descriptive statistics are reported by the group's mean and standard error.

## Results

### Experiment 1

On average, the hotspot of the OP was positioned at 5.43±0.65 cm (mean ± SD) lateral from the vertex and 0,29±0,83 cm anteriorly to the intra-aural line. The RMT was 43%±5.25 of the maximum stimulation output.

Subjects recognized the weight of the object lifted in the videos with high accuracy in the single task (100% correct) and dual task conditions (98±1% correct) which were not significantly different (t(12) = 1.4, p = 0.186). The auditory discrimination task was challenging and discrimination accuracy was 71% across all movie conditions. Importantly, accuracy in the baseline condition (70.8±3.7%) did not differ significantly from the LIGHT (69.6±4.8%) or HEAVY condition (72.2±3.9%) (F(2,20) = 0.179 p = 0.83).

**Figure 2:**
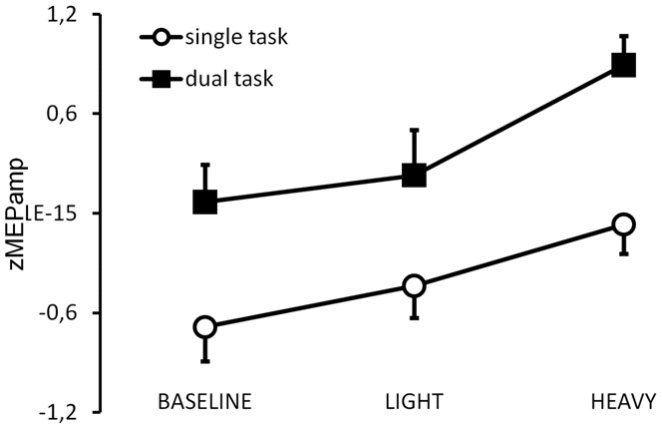
Interaction effect of the observed weight and the auditory discrimination task on MEP values of the OP in experiment 1. z transformed MEP amplitudes (zMEPamp) are shown for the single task (open circles) compared with the dual task (black squares) for the three different observation conditions. Vertical bars indicate standard errors.

Corticomotor excitability of the OP was modulated by MO and in a weight-dependent manner, such that zMEPamp were smallest for the baseline condition and highest for the HEAVY movie condition (figure 2). Importantly, this modulation was similar when the movies were observed as a single or a dual task, as indicated by a significant main effect of Movie (F(2, 26) = 4.5534, p = .021) in the absence of a significant Movie x Task interaction (F(2, 26) = .28428, p = .75). This result was further confirmed by pre-planned comparisons revealing that the zMEPamp were significantly larger for the HEAVY than the LIGHT movies irrespective of whether MO was performed as single (t(13) = 1.82323, p = .047) or dual task (t(13) = 2.39015, p = .017). However, performing the auditory discrimination task enhanced zMEPamp across all movies compared to the single task conditions as indicated by a highly significant Task main effect (F(1,13) = 17.769, p<.005).

The pre-trigger EMG exhibited small but significant differences such that higher values were found for the baseline (0.0019±0.0004 RMSE EMG) than the other conditions (LIGHT: 0.00181±0.0004; HEAVY: 0.00186±0.0004), as indicated by a significant Movie effect (F(2, 26) = 4.8563, p = .017). No other effects reached significance (F(1, 13)≤3.8261, p≥.074).

### Experiment 2

On average, the hotspot of the OP was positioned at 5.04±0.47 cm (mean ± SD) lateral from the vertex and 0,36±0,81 cm anteriorly to the intra-aural line. The RMT was 41,63%±6,02 of the maximum stimulation output and was not significantly different from experiment 1.

Also in experiment 2 subjects performed MO with high accuracy for both the single (99.3±0.5% correct) and the dual task conditions (99.7±0.3% correct) which were not significantly different (t(10) = 1, p = 0.3). The visual discrimination task was performed with 80% accuracy which did not differ across the movie conditions (baseline: 83±0.05%, LIGHT: 80±0.04%, HEAVY: 78±0.05%) (F(2, 20) = .92, p = .41).

**Figure 3:**
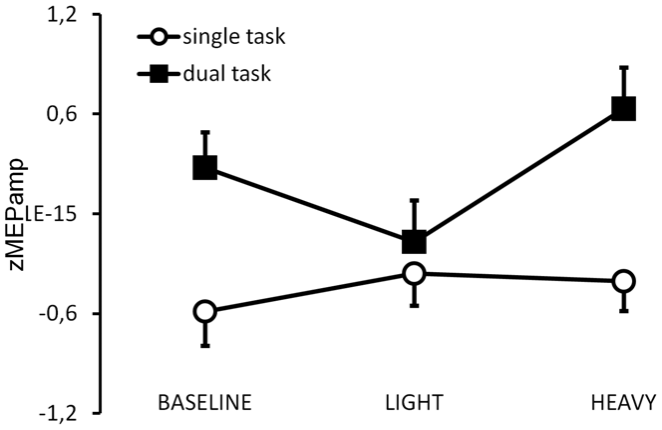
Interaction effect of the observed weight and the visual discrimination task on MEP values of the OP in experiment 2. z transformed MEP amplitudes (zMEPamp) are shown for the single task (open circles) compared with the dual task (black squares) for the three different observation conditions. Vertical bars indicate standard errors.

For the zMEPamp (figure 3), no significant effects were found for the Movie (F (2, 20)  = .88511; p = .42823) or the Movie x Task interaction (F (2, 20)  = 1, 4389; p = .26072). Particularly the fact that zMEPamp responses were not higher for the HEAVY than the LIGHT single task condition was unexpected and inconsistent with our hypothesis. For the dual-task condition, preplanned comparisons revealed that zMEPamp was significantly larger for the HEAVY as compared to the LIGHT movie (t(11) = 2.24, p = 0.023), however, this effect was largely driven by the low response to the LIGHT dual task condition. By contrast, zMEPamp did not differ between the dual task HEAVY and the dual task baseline condition (t(11) = 1.33, p = 0.10). Thus, the surprising result for experiment 2 was that there was no consistent Movie effect. A power analysis revealed that we would have to increase sample size of experiment 2 to n = 134 to find an effect of similar size as in experiment 1 with only 14 subjects (where we had a power of 71%). This confirms that the movies were less effective in modulating zMEPamp when combined with a visual than with an auditory discrimination task. As in experiment 1, there was a strong Task effect such that zMEPamp values were significantly larger in the dual than the single task condition (main effect (F (1, 10)  = 27,942; p<.005). This effect was mainly driven by the baseline and the HEAVY condition (t(11)≥3.28, p<0.005).

The pre-trigger EMG was small and differed only slightly across conditions as indicated by non significant statistics (F (2, 20) < = .30380; p> = .77272).

## Discussion

In this study we used a dual-task approach to explore whether Movement Observation is an attention-demanding process and whether attentional costs depend on the sensory modality accessed by the secondary task. Our novel finding was that experiment 1 yielded no evidence for dual-task interference when MO was performed together with an auditory discrimination task as neither discrimination accuracy in the secondary task nor the action specific modulation of M1 excitability in response to MO differed between single and dual-task conditions. By contrast, experiment 2 revealed that MO specific modulation of M1 excitability was perturbed when the visual discrimination cues were added to the MO videos. Notably, TMS parameters were very similar across experiments such that the differential outcomes of experiment 1 and 2 resulted most likely from the auditory versus visual discrimination task. Together our findings indicate that dual task interference might arise when competing visual stimuli are present (experiment 2), but not when the competing task accesses the auditory domain (experiment 1).

### No dual-task interference when MO was performed together with a cross-modal task

Experiment 1 revealed no evidence that performing MO in parallel with an auditory discrimination task caused dual-task interference. More specifically, there were no differences in discrimination accuracy when the auditory discrimination task was performed while observing the LIGHT or HEAVY movies or during the baseline condition, i.e., when subject saw a static image and could completely focus on the secondary task. This result is not surprising as the MO task was chosen such that it had low demands as the force requirements were recognized easily and with nearly 100% accuracy. Despite that MO was performed effortless, it affected OP excitability in a force related manner, such that MEP amplitudes were significantly higher when observing the HEAVY than the LIGHT movie. For the single task condition this is consistent with previous work [9], [10] and in line with the notion that that the observer's motor system directly matches the perceived action to the corresponding motor representation [13], [45]. Importantly, the same modulation of M1 excitability in response to the HEAVY versus LIGHT movie was found when MO was performed under dual-task conditions, indicating that observation-execution matching was still intact and that the force requirements were represented in the observer's motor system. Together, the behavioral and neural results strongly suggest that there was no dual-task cost when the auditory discrimination task was executed simultaneously with MO supporting the notion that movement observation is a near automatic process.

One explanation for this finding was proposed by Alais et al. (2006) who suggested that each sensory modality is entitled to its own separate attention resource and that dual-task interference is less likely to occur when different modalities are accessed. According to the capacity sharing model of Navon and Gopher (1979) this suggests that the attentional demands of MO were low, not exceeding computational resources at the central level. This is consistent with previous research demonstrating that imitation might emerge involuntarily suggesting that observation-execution matching during MO is an automatic process [20]–[25], [32].

### Visual distracters influence M1 responses to MO

Even though the weight perceptions as well as the visual discrimination task were performed very accurately in experiment 2, MEP results were highly unexpected. Contrary to our hypothesis, experiment 2 revealed only inconsistent weight-related modulations of M1 excitability. Particularly in the single task condition, MEP responses were not higher for the HEAVY than the LIGHT condition which was against our expectations based on previous research [10]. For the dual-task condition, we found a significantly lower response to the LIGHT than the HEAVY videos, but no significant differences between the HEAVY and the baseline condition. This is again inconsistent with previous research which predicted that the MEP amplitudes should be significantly larger for MO than for a control condition not showing motion stimuli [8]–[19].

The lack of consistent MO effects in our present study was most likely caused by adding additional visual cues to the motor action. One explanation is that the blinking frame exerted a strong influence on M1 excitability via indirect anatomical pathways. Thus, the observer's motor system might have reflected the motor information shown in the video, but this effect was masked because excitability was additionally influenced by the changing colour stimuli of the rim. Even though there is no direct anatomical connection between visual cortex and M1, visual areas project to parieto-temporal regions which are connected to premotor areas that, in turn, project to M1[46], [47]. For example, the STS region, which is assumed to provide the main visual input to the mirror neuron system, responds to a large variety of different visual stimuli [23]. It was shown previously that the presence or absence of visual input has an effect on M1 excitability [48], however, currently there is no evidence that differences in colour would have a similar effect [49]. Moreover, it has to be noted that the TMS pulse was always applied in the short pause between the two series of changing colours, such that the rim colour was black in all conditions. Thus, even though we cannot firmly exclude that the changing colours influenced M1 excitability, this explanation seems to be less likely and is currently not supported by the literature.

An alternative explanation for our unexpected results is that the changing colours might have acted as distracters, which provoked involuntary gaze shifts. Particularly, as subjects might have guessed the weight of the object after the first trial shown in a block. One has to note that subjects were repeatedly and explicitly reminded to look at the hand-object interaction shown at the videos and that TMS stimulation was provided during the break between the two series of color cues, i.e. when the screen was black and no information for the color discrimination task was provided. However, we can only assume that subjects complied with this instruction and looked at the MO stimuli during TMS application because it was not possible to measure eye movements in the present experiment. Moreover, in a previous study showing similar movements as used here, subjects were required to look at a fixation point which was either located slightly above or slightly below the effector-object interaction [24]. Even though subjects did not look at the action directly, this study reports strong brain activation within classical mirror-neuron areas in the inferior parietal and inferior frontal/ventral premotor cortex suggesting that also non-foveal vision might be sufficient to induce activity ”resonating” with the observed movement. However, future studies will have to resolve the issue whether resonating behavior in M1 depends on foveal vision during MO.

Another, even though not mutually exclusive, explanation is that the changing colours of the rim induced shifts of visual attention, either overt or covert.

Such bottom-up mechanisms have been described previously, such that environmental cues as the colour changes of the rim drawn the observer's attention involuntarily to a new position in space [50]–[52]. Thus, the presence of distracting visual stimuli might have perturbed weight-dependent M1 responses as typically observed when the same movement stimuli are observed without additional visual distracters (see experiment 1 and [9], [10]). This interpretation is consistent with the findings of Bach et al. (2007), Muthukumaraswamy and Singh (2008) and Chong et al. (2008) who argued that visual attention needs to be directed towards the relevant stimulus to increase the efficiency of MO.

Note however, that movement is a strong exogenous cue that attracts attention nearly unconsciously [53], [54]. This feature inherent to most stimuli used for MO (except when still pictures are shown) might contribute to the automatic imitation effects reported earlier when biological motion [30], [55]–[57] or even non-biological motion stimuli were observed [58]–[61].

Finally, one has to note that excitability changes in M1 are reflecting many different features of the observed movements. For example, M1 activity mirrors not only times the course of the observed action but exhibits also anticipatory activity because future actions or goals can be simulated in the observers motor system [17], [62]–[64] . Similarly, M1 encodes not only muscle and force related aspects but is additionally also influenced by the compatibility of the observer's and model's posture [14], [17], [18]. Even though these aspects were kept constant across our experimental conditions, these previous findings confirm that M1 excitability is a compound measurement reflecting many different features related to the observed stimuli. Note also that weight perception was very accurate even though MEP responses revealed no consistence result pattern. Thus, it is possible that other factors then the observed hand-object interaction influenced corticomotor excitability in experiment 2.

M1 receives projections deriving from different upstream areas with the inferior frontal gyrus/ventral premotor cortex (PMv) [65], being probably the most important input area. PMv has been shown to encode kinematics and motor aspects of the observed action [66] and transiently disrupting PMv impairs (1) perceptual weight-judgment tasks [67] as well as (2) M1 responses to simple biological movements as measured by corticomotor excitability [65]. This suggests that PMv plays a causal role in MEP facilitation during MO. Neurons in PMv exhibit not only motor or mirror properties, but many respond also to purely visual input which was mostly demonstrated in the context of object properties [68]. As such, PMv might have been activated by the colored rim, influencing M1 excitability also for non-motion related visual information.

### Discrimination tasks increase M1 excitability

We found that OP excitability in M1 was substantially increased during the discrimination task. This result was unexpected and has, to our best knowledge, not been described in the TMS literature previously. However, the effect was statistically very robust and observed across both experiments. Note also that the sensory stimuli were identical in the single and dual task condition, indicating that the increase of M1 excitability rather resulted from cognitive demands due to discrimination than the sensory input per se. Using functional magnetic resonance imaging, increased brain activation was demonstrated for dual-tasking [69] as well as cognitive demanding tasks [34]. Therefore, the increased M1 excitability could be explained by a higher activation of areas upstream from M1 which might have been directly involved in the increased working memory load from the discrimination task or by a general increase in arousal.

Another potential explanation was yielded by Van Leeuwen et al (2009) who showed that priming effects induced by biological motion stimuli were stronger when working memory load was high while no such effects were found when responding to spatial cues unrelated to body movements [70]. Using a similar paradigm, Gowen at al. (2010) argued that automatic imitation in response to biological versus non-biological primes depends on top-down attentional control mechanisms and that visual manipulations, like a flash created by a yellow rim, are sufficient interact with the attentional focus making it either too narrow or too diffuse [71]. Importantly, the results of Van Leeuwen et al (2009) indicate that an increase of central work load might interfere with executive functions necessary to inhibition overt imitation. At a general level, our results are in line with these previous findings because the increased excitability in the dual-task condition might be indicative of an overall dis-inhibition of the motor system.

Alternatively, our data might be explained by recent findings that the excitability of hand muscles increases when the order of a series of elements needs to be processed as during counting [72]. This was indeed the case in our study and might have lead to the increase of M1 excitability during the discrimination task.

### Conclusions and potential clinical applications

In summary our data show that MO activated action representations in M1 effectively when subjects performed a secondary task not accessing the visual modality. By contrast, MO specific facilitation of the observer's M1 was perturbed when visual distracters were added to the motor action stimuli, even when subjects were instructed to ignore these additional cues. This indicates that MO per se makes little demands on computational resources involved in attentional control at a central level. However, MO might be impaired when competing visual stimuli are present causing structural interference within visuo-motor processing pathways [37], [73] or interfering with attentional control either via bottom-up or top-down mechanisms. This might be particularly the case if saliency is higher for non-motor than motor information represented in the visual stimuli.

Our findings might have practical implications for using MO in rehabilitation settings, for example after stroke. During recent years, there is increasing theoretical and empirical support that cognitive strategies such as MO, imitation or motor imagery are valuable additions to physical therapy in stroke rehabilitation [40]. Most empirical studies have investigated the effect of motor imagery which was shown to be beneficial in augmenting physical therapy in acute [74] subacute [75] and chronic stroke [76], [77] patients. However, motor imagery requires voluntary mental effort and, in particular, a high degree of attentional control. Therefore, MO which is a task with very low cognitive demands might be a valuable alternative that could be used already in the acute phase after the incident or in patients suffering from attentional deficits.
